# Vitamin D Metabolites and Binding Protein Predict Preeclampsia in Women with Type 1 Diabetes

**DOI:** 10.3390/nu12072048

**Published:** 2020-07-10

**Authors:** Clare B. Kelly, Carol L. Wagner, Judith R. Shary, Misti J. Leyva, Jeremy Y. Yu, Alicia J. Jenkins, Alison J. Nankervis, Kristian F. Hanssen, Satish K. Garg, James A. Scardo, Samar M. Hammad, Christopher E. Aston, Timothy J. Lyons

**Affiliations:** 1 Division of Endocrinology, Medical University of South Carolina, Charleston, SC 29425, USA; clare.kelly@kcl.ac.uk (C.B.K.); leyva@musc.edu (M.J.L.); yuje@musc.edu (J.Y.Y.); alicia.jenkins@ctc.usyd.edu.au (A.J.J.); 2 Department of Women and Children’s Health, King’s College London, London SE1 7EH, UK; 3 Division of Neonatology, Medical University of South Carolina, Charleston, SC 29425, USA; wagnercl@musc.edu (C.L.W.); sharyj@musc.edu (J.R.S.); 4 University of Sydney, NHMRC Clinical Trials Centre, Camperdown, Sydney, NSW 1450, Australia; 5 Royal Women’s Hospital, Melbourne, VIC 3052, Australia; alison.nankervis@mh.org.au; 6 Department of Endocrinology, Oslo University Hospital and Institute of Clinical Medicine, University of Oslo, 0424 Oslo, Norway; k.f.hanssen@medisin.uio.no; 7 Barbara Davis Center for Childhood Diabetes, University of Colorado, Denver, CO 80045, USA; satish.garg@ucdenver.edu; 8 Spartanburg Regional Medical Center, Spartanburg, SC 29303, USA; scardo@charter.net; 9 Department of Regenerative Medicine and Cell Biology, Medical University of South Carolina, Charleston, SC 29425, USA; hammadsm@musc.edu; 10 The Department of Pediatrics, University of Oklahoma Health Sciences Center, Oklahoma City, OK 73104, USA; chris-aston@ouhsc.edu; 11 Wellcome-Wolfson Institute for Experimental Medicine, Queen’s University Belfast, Belfast BT9 7BL, UK

**Keywords:** preeclampsia, pregnancy, type 1 diabetes, vitamin D, vitamin D binding protein, 25-hydroxyvitamin D, 1,25-dihydroxyvitamin D

## Abstract

The risk for preeclampsia (PE) is enhanced ~4-fold by the presence of maternal type 1 diabetes (T1DM). Vitamin D is essential for healthy pregnancy. We assessed the total, bioavailable, and free concentrations of plasma 25-hydroxyvitamin D (25(OH)D), 1,25-dihydroxyvitamin D (1,25(OH)_2_D), and vitamin D binding protein (VDBP) at ~12, ~22, and ~32 weeks’ gestation (“Visits” (V) 1, 2, and 3, respectively) in 23 T1DM women who developed PE, 24 who remained normotensive, and 19 non-diabetic, normotensive women (reference controls). 25(OH)D deficiency was more frequent in diabetic than non-diabetic women (69% vs. 22%, *p* < 0.05), but no measure of 25(OH)D predicted PE. By contrast, higher 1,25(OH)_2_D concentrations at V2 (total, bioavailable, and free: *p* < 0.01) and V3 (bioavailable: *p* < 0.05; free: *p* < 0.01), lower concentrations of VDBP at V3 (*p* < 0.05), and elevated ratios of 1,25(OH)_2_D/VDBP (V2, V3: *p* < 0.01) and 1,25(OH)_2_D/25(OH)D (V3, *p* < 0.05) were all associated with PE, and significance persisted in multivariate analyses. In summary, in women with T1DM, concentrations of 1,25(OH)_2_D were higher, and VDBP lower, in the second and third trimesters in women who later developed PE than in those who did not. 1,25(OH)_2_D may serve as a new marker for PE risk and could be implicated in pathogenesis.

## 1. Introduction

Adequate levels of vitamin D are essential for bone health, immune function, proliferation and differentiation of cells, inflammation, insulin secretion and action, and vascular health [1]. Vitamin D deficiency is common worldwide, involving genetic, lifestyle and geographical factors [1,2]. Vitamin D metabolism is markedly altered during pregnancy. Specifically, for reasons not fully understood, the plasma concentrations of the active metabolite 1,25-dihydroxyvitamin D (1,25(OH)_2_D) increase 2–3 fold during the first trimester, reaching concentrations that would normally be toxic, and continue to increase as pregnancy advances [3,4,5]. Associations of 1,25(OH)_2_D levels with preeclampsia (PE) in the presence of maternal diabetes have not been investigated. Of interest, in a rat model, treatment with 1,25(OH)_2_D was shown to ameliorate preeclampsia [6].

In the general population, the plasma concentration of 25-hydroxyvitamin D (25(OH)D), the prohormone and precursor of 1,25(OH)_2_D, is considered the principal metric for assessing vitamin D status: deficiency and insufficiency are defined as <20 and <32 ng/mL (<50 and <80 nmol/L), respectively [7]. Vitamin D deficiency is associated with poor pregnancy outcomes for both mother and child [2,8,9,10,11,12,13,14,15,16,17,18,19,20,21]. Both 25(OH)D and 1,25(OH)_2_D can be measured in plasma as total, bioavailable, and free forms; both metabolites are highly lipophilic and, in plasma, are almost entirely protein-bound. Vitamin D binding protein (VDBP) is the principal carrier of both 25(OH)D and 1,25(OH)_2_D (85–90% of the total); a smaller amount of each metabolite (10–15%) is bound to albumin (a lower affinity carrier), and ≤1% exists unbound in the “free” form [22,23]. “Bioavailable” vitamin D is the sum of unbound (free) and albumin-bound forms: it is considered physiologically relevant because the affinity of vitamin D for albumin is so low [22,23,24,25,26].

PE is a multisystem disorder defined by hypertension and proteinuria or other end-organ dysfunction, with onset after 20 weeks’ gestation in a previously normotensive woman [27]. Women with type 1 diabetes (T1DM) have a markedly increased risk for PE (~20% vs. ~5% in the general population) [28]. Vitamin D deficiency is associated with abnormal placentation, altered angiogenesis, immune dysfunction, abnormal insulin secretion and action, adverse lipid profiles, and inflammation: problems that are also associated with diabetes [2,4,11,13,14,15,16,17,19,29]. Vitamin D deficiency may also be implicated in PE [9,13].

We previously reported associations between PE and the concentrations of fat-soluble vitamins and antioxidant pro-vitamins in women with T1DM [30]. In that study, we used only one measure of vitamin D status, 25(OH)D, measured by HPLC. Women with T1DM were more likely to be vitamin D deficient than those without diabetes: indeed almost all the diabetic women were deficient, and the concentrations did not differ significantly according to subsequent PE status [30]. Extending that work, we now investigate whether total, bioavailable, or free forms of 25(OH)D; its active metabolite 1,25(OH)_2_D; VDBP concentrations; and relevant ratios are associated with the risk for subsequent PE in women with T1DM. As before, we include a group of healthy, normotensive, non-diabetic pregnant women to obtain normal reference values.

## 2. Research Design and Methods

### 2.1. Study Design and Participants

Participants in the current study are a subset of the “Markers and Mechanisms for PreEclampsia in Type 1 Diabetes” (MAMPED) cohort. The MAMPED design, participant characteristics, and inclusion/exclusion criteria have been described previously [30,31,32,33,34,35,36]. The overall goal of MAMPED was to identify early markers and potential mechanisms for PE in the context of pregnancy complicated by maternal T1DM. Briefly, MAMPED was a longitudinal, prospective pregnancy study of 151 women with T1DM, with 24 non-diabetic women as reference controls: all were enrolled in the first trimester and followed until delivery. The study was conducted in Norway, Australia, and the United States, and the participants were predominantly Caucasian (86%). All were free of hypertension, proteinuria, and microalbuminuria at enrolment (urinary albumin/creatinine ratio <30 mg/g at the first study visit when the gestational age was 9–16 weeks). PE was defined as new-onset hypertension (>140/90 mmHg) and proteinuria (>300 mg/24 h) after 20 weeks’ gestation. Clinical data and specimens (plasma and urine) were collected at three visits: in the first trimester (V1: gestation 12.2 ± 1.9 w (mean ± SD)), mid-second trimester (V2: 21.6 ± 1.5 w), and early third trimester (V3: 31.5 ± 1.7 w). Samples were stored at −80 °C until analysis. The study was conducted according to Declaration of Helsinki guidelines and approved by the Institutional Review Boards at all participating institutions. Written informed consent was obtained from all participants.

In accordance with the original MAMPED design, in the primary analysis, type 1 diabetic women with PE were compared with a matched group (matched by age, diabetes duration, and parity) without PE. For this report, these groups comprised 23 (of the original 26) who developed PE (DM+PE+; three were unavailable through sample attrition), and 24 who remained normotensive (DM+PE−; from an original matched subset of 26). A secondary analysis compared the DM+PE− group with the reference control group of non-diabetic, non-PE women (DM−). All the study visits occurred prior to the onset of PE.

Medication usage was recorded at V1. All women with diabetes were taking insulin. A majority were taking folic acid at V1 (DM+PE+: 70%; DM+PE−: 71%; DM−: 42%, *p* > 0.05), but overall, a minority took vitamin supplements (DM+PE+: 39%; DM+PE−: 50%; DM−: 32%, *p* > 0.05). The use of vitamin supplements did not differ by diabetes status, PE outcome, or vitamin D deficiency.

### 2.2. Laboratory Measures

Plasma 25(OH)D concentrations were measured using the DiaSorin Corporation 25-hydroxyvitamin D ^125^I RIA Kit (Stillwater, MN, USA). No dilution was required. The intra- and inter-assay coefficients of variation were ≤10%. 1,25(OH)_2_D was measured by a Quantitative Chemiluminescent Immunoassay at ARUP laboratories, Salt Lake City, UT, after a 6.25-fold dilution. VDBP was measured using the human Vitamin D BP Quantikine ELISA kit (R&D systems, Minneapolis, MN, USA), according to the manufacturer’s protocol: plasma samples were diluted 10,000-fold and assayed in duplicate, and the intra- and inter-assay coefficients of variation were 3% and 7%, respectively. The operators were masked to clinical status and sample order throughout, and all the samples from an individual were run in the same assay. Circulating albumin levels were measured at the Department of Clinical Biochemistry, Royal Victoria Hospital, Belfast, Northern Ireland. The levels of free and bioavailable 25(OH)D and 1,25(OH)_2_D were calculated from the measured total 25(OH)D, 1,25(OH)_2_D, VDBP, and albumin concentrations using the following equations [22,23,37].

$$\mathrm{Calculated}\;\mathrm{free}\;\left[D\right]=\frac{\mathrm{Total}\;\left[D\right]}{1+\left(K_{Alb}\times \left[\mathrm{Albumin}\right]\right)+\left(K_{VDBP}\times \left[\mathrm{VDBP}\right]\right)}$$

$$\mathrm{Bioavailable}\;\left[D\right]=\left(K_{Alb}\times \left[\mathrm{Albumin}\right]+1\right)\times \mathrm{calculated}\;\mathrm{free}\;[D]$$

$$\mathrm{The}\;\mathrm{percentage}\;\mathrm{of}\;\mathrm{free}\;\left[D\right]=\frac{\mathrm{Free}\;\left[D\right]}{\mathrm{Total}\;\left[D\right]}$$

$$\left[D\right]={25(\mathrm{OH})D\;\mathrm{or}\;1,25(\mathrm{OH})}_{2}D$$

[Albumin]=serum albumin in g/L ÷ 66,430 g/mol

[VDBP]=serum VDBP in g/L ÷ 58,000 g/mol

Note that the affinity constants for albumin (25(OH)D: K_Alb_ = 6 × 10^5^ M^−1^; 1,25(OH)_2_D: K_Alb_ = 5.4 × 10^4^ M^−1^) are substantially lower than those for VDBP (25(OH)D: K_VDBP_ = 7 × 10^8^ M^−1^; 1,25(OH)_2_D: K_VDBP_ = 4 × 10^7^ M^−1^) [22,23].

The amount of each vitamin D metabolite bound to VDBP was calculated by subtracting the bioavailable from the total vitamin D. Using this information, it was possible to calculate the VDBP saturation for 25(OH)D and 1,25(OH)_2_D.

### 2.3. Statistical Analysis

**Figure 1:**
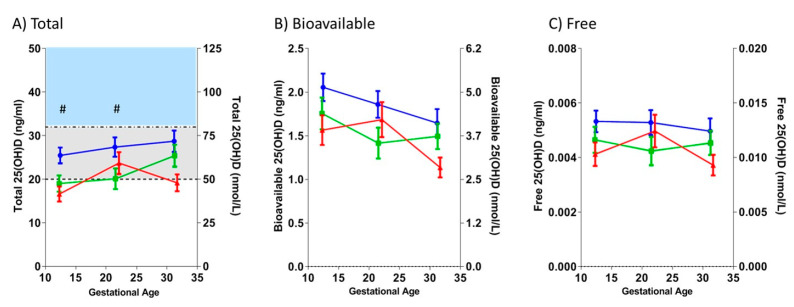
Maternal concentrations of total, bioavailable, and free 25-hydroxyvitamin D (25(OH)D), according to type 1 diabetes (T1DM) and/or preeclampsia (PE) status. Longitudinal changes in (**A**) total, (**B**) bioavailable, and (**C**) free 25(OH)D concentrations, prior to the clinical onset of PE. Values (mean ± SEM) are plotted against the three study visits, corresponding to ~12, ~22, and ~32 gestational weeks, respectively. Red: women with T1DM who subsequently developed PE (DM+PE+); green: women with T1DM who did not develop PE (DM+PE−); blue: non-diabetic, normotensive women (DM−). ^#^ *p* < 0.05 for DM+PE− vs. DM−. Cut points for Total 25(OH)D: Normal: >32 ng/mL (**shaded blue**), Insufficient: ≥20 and ≤32 ng/mL (**shaded grey**), Deficient: <20 ng/mL.

**Figure 2:**
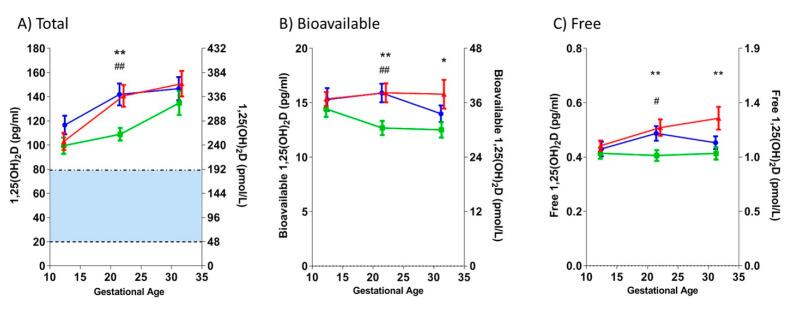
Maternal concentrations of total, bioavailable, and free 1,25-dihydroxyvitamin D (1,25(OH)_2_D), according to T1DM and/or PE status. Longitudinal changes in (**A**) total, (**B**) bioavailable, and (**C**) free 1,25(OH)_2_D concentrations, prior to the clinical onset of PE. Values (mean ± SEM) are plotted against the three study visits, corresponding to ~12, ~22, and ~32 gestational weeks, respectively. Red: women with T1DM who subsequently developed PE (DM+PE+); green: women with T1DM who did not develop PE (DM+PE−); blue: non-diabetic, normotensive women (DM−). * *p* < 0.05, ** *p* < 0.01 for DM+PE− vs. DM+PE+; ^#^ *p* < 0.05, ^##^ *p* < 0.01 for DM+PE− vs. DM−. Non-pregnant normal range for Total 1,25(OH)_2_D (based on ARUP Laboratories, for the “general population”): 19.9–79.3 pg/mL (shaded blue).

As pre-defined in MAMPED, the primary analysis compared DM+PE+ with DM+PE−. Secondary analyses compared “uncomplicated” T1DM women (DM+PE−) with non-diabetic women (DM−). The results are expressed as means ± SDs (Table 1 and Table 2) or SEMs (Figure 1 and Figure 2). Groups were compared using unpaired Student’s t tests for continuous measures and $\chi^{2}$ tests for categorical measures; unpaired tests were used because of the differential sample attrition. Analyses of repeated measures used Friedman’s test. Logistic regression, with and without covariate adjustments, was used to estimate the probability of women with T1DM developing PE based on clinical characteristics and biomarker values. The following covariates were selected based on differences at the time of visit and/or their known associations with vitamin D metabolism: BMI, glycated hemoglobin (HbA1c), and total adiponectin [38]. All the tests were two-tailed, with *p* < 0.05 considered significant. Statistical analyses were performed using the SPSS software, version 22 (IBM Corp, Armonk, NY, USA).

## 3. Results

*Maternal characteristics:*Table 1 shows the baseline clinical characteristics of all the women. There were no significant differences in age, ethnicity, smoking, gravidity, parity, duration of T1DM, systolic and diastolic blood pressure, mean arterial pressure (MAP), total cholesterol, LDL cholesterol, triacylglycerols, and gestational age per visit between DM+PE+ and DM+PE−. However, at the initial study visit, the HbA1c, Body Mass Index (BMI), and total daily insulin dose were significantly higher in DM+PE+ than in DM+PE−, and HDL cholesterol was significantly lower. There were no significant differences between the two normotensive groups at V1, except, as expected, HbA1c was higher in women with diabetes.

*25(OH)D deficiency was not associated with subsequent PE in women with T1DM:* Vitamin D insufficiency and deficiency are defined as concentrations <32 and <20 ng/mL, respectively [7]. As shown in Figure 1, a majority (97%) of all the women fell below the “normal” level of vitamin D throughout pregnancy. Women with T1DM were more likely to be 25(OH)D deficient (DM+PE+: 73%; DM+PE−: 65%) than non-diabetic women (22%) at the first visit (*p* = 0.009); however, there were no significant differences in any measure of 25(OH)D during pregnancy between the DM+PE+ and DM+PE− groups. Total 25(OH)D was lower in DM+PE− than in DM− groups at the beginning of pregnancy (V1, *p* = 0.020; V2, *p* = 0.034), but neither bioavailable nor free 25(OH)D differed by diabetes status at any visit.

*Higher second and/or third trimester 1,25(OH)_2_D concentrations (total, bioavailable, and free) are associated with subsequent PE in women with T1DM:* As shown in Figure 2, in the DM+PE+ vs. the DM+PE− group, the total 1,25(OH)_2_D was higher at V2 (*p* = 0.005), and bioavailable and free 1,25(OH)_2_D were higher at V2 and V3 (bioavailable: V2, *p* = 0.005; V3, *p* = 0.031; free: V2, *p* = 0.007 and V3, *p* = 0.009). In the DM+PE− vs. the DM− group, all measures of 1,25(OH)_2_D were lower at V2 (total, *p* = 0.002; bioavailable, *p* = 0.004; free, *p* = 0.018). Total 1,25(OH)_2_D significantly increased as pregnancy advanced in DM+PE+ (*p* < 0.001) and DM+PE− (*p* = 0.007).

Using logistic regression, the data were analyzed without and with covariates to assess the effectiveness of 1,25(OH)_2_D as a biomarker of PE. At V2, without covariates and in women with T1DM only, every 1 pg/mL increase in total 1,25(OH)_2_D increased the odds of developing PE by 3% (OR: 1.03 (1.01–1.05), *p* = 0.012), while every 1 pg/mL increase in bioavailable 1,25(OH)_2_D increased the odds for PE by 28% (OR: 1.28 (1.06–1.54), *p* = 0.010). Likewise, at V3, every 1 pg/mL increase in bioavailable 1,25(OH)_2_D increased the odds for PE by 18% (OR: 1.18 (1.00–1.39), *p* = 0.047). Covariate analyses including BMI, HbA1c, and total adiponectin did not affect the significance.

*Ratios of total, bioavailable, and free 1,25(OH)_2_D to corresponding 25(OH)D concentrations* (Table 2)*:* In the DM+PE+ vs. the DM+PE− group, the total, bioavailable, and free 1,25(OH)_2_D/25(OH)D (product/substrate) ratios were all higher at V3 (all *p* < 0.05). There were no significant differences in these ratios at any stage of pregnancy between the DM+PE− and DM− groups, and there were no significant changes over time in any of the groups. For women with T1DM only, at V3, for every unit increase in total 1,25(OH)_2_D/25(OH)D, the odds of developing PE increased by 17% (OR: 1.17 (1.01–1.35), *p* = 0.037); however, this significance did not persist after covariate adjustment.

*Lower VDBP and higher 1,25(OH)_2_D/VDBP ratio are associated with subsequent PE in women with T1DM:* As summarized in Table 2, in the DM+PE+ vs. the DM+PE− group, VDBP was lower at V3 (*p* = 0.032), and total 1,25(OH)_2_D/VDBP and [1,25(OH)_2_D bound to VDBP]/VDBP were both higher at V2 and V3 (all *p* < 0.01). Total 25(OH)D/VDBP and [25(OH)D bound to VDBP]/VDBP did not differ between DM+PE+ and DM+PE− at any study visit. In the DM+PE− vs. the DM− group, the total 1,25(OH)_2_D/VDBP and [1,25(OH)_2_D bound to VDBP]/VDBP were lower at V2 (*p* = 0.025 and *p* = 0.018, respectively). VDBP significantly increased throughout pregnancy in all groups (*p* < 0.001). For women with T1DM only, for every 1 mg/dL increase in VDBP at V3, the odds of developing PE decreased by 8% (OR: 0.92 (0.85–1.00), *p* < 0.05). At V2, for every unit increase in total 1,25(OH)_2_D/VDBP, the odds of developing PE increased almost three-fold (OR: 2.71 (1.28–5.77), *p* = 0.009). Likewise, at V3, for every unit increase in this ratio, the odds of developing PE increased similarly (OR: 2.53 (1.21–5.29), *p* = 0.013). The consideration of covariates had no effect.

## 4. Discussion

*Main findings:* This longitudinal study of pregnancy in T1DM is the first to report multiple detailed measures of vitamin D (total, bioavailable, and free concentrations of 25(OH)D and 1,25(OH)_2_D; VDBP; and relevant ratios) and their associations with subsequent PE. We were surprised to find that at V2 and V3, elevated plasma 1,25(OH)_2_D, low VDBP, and elevated 1,25(OH)_2_D/VDBP ratios were associated with subsequent PE.

By contrast, 25(OH)D, the standard metric for vitamin D, did not predict PE; of note, however, insufficient or deficient levels were almost universal in our diabetic cohort. Reported associations of vitamin D deficiency in diabetic pregnancy include preterm birth, increased T1DM rates in offspring of women with T1DM, and poor glycemic control [12,17,20,21,29]. Some studies in non-diabetic pregnant women have suggested that low total 25(OH)D is associated with contemporaneous [10] or subsequent PE [9,12], perhaps limited to early-onset disease [13]. Our present finding of no significant association is consistent with our previous study (that used different methodology to measure 25(OH)D) [30] and with a study by Vestgaard et al. [17]. The present work extends those findings, showing that neither bioavailable nor free forms of 25(OH)D predicted PE, with the caveat that our cohort did not contain vitamin D-sufficient women.

Maternal 1,25(OH)_2_D concentrations are known to increase dramatically throughout normal pregnancy [39], reaching levels otherwise considered toxic; the reasons are not fully understood. Studies of 1,25(OH)_2_D throughout pregnancy are sparse; none address maternal diabetes. A small longitudinal case-control study of PE in non-diabetic women (10 PE cases vs. 40 controls) found no association between serum 1,25(OH)_2_D in the late second and early third trimesters and subsequent PE [8]. By contrast, we found that bioavailable and free 1,25(OH)_2_D both predict PE at the second and early third trimesters, while total 1,25(OH)_2_D predicted PE at the second trimester. The pregnancy-associated increase in 1,25(OH)_2_D is known to be accompanied by, but disproportionate to, an increase in VDBP, and a change in binding affinity has been suggested [39,40]. High 1,25(OH)_2_D is thought to reflect the calcium needs of the developing fetus, increasing calcium absorption and up-regulating trans-placental transport [40].

Lower VDBP concentrations were predictive of PE at the third trimester, and the ratio of 1,25(OH)_2_D/VDBP was predictive at V2 and V3. A larger study is needed to determine whether this ratio is a better predictor than 1,25(OH)_2_D alone. VDBP, like many plasma proteins, significantly increased as pregnancy advanced, independently of diabetes or PE status.

*Strengths and Limitations:* This longitudinal study of pregnant women with T1DM is unique in its detailed measures—not only of the total, bioavailable, and free concentrations of 25(OH)D and 1,25(OH)_2_D but also of VDBP—and in relating these data to (late-onset) PE. It therefore addresses significant gaps that have recently been highlighted in the literature [41]. Although the study cohort was small, it was rigorously phenotyped and was free of proteinuria and hypertension at enrolment. The gestational time-points were well-defined, and all study visits occurred prior to PE onset. A non-diabetic control group provided reference values for normal pregnancy.

The limitations include the reliance on fixed affinity constants to estimate the free forms of vitamin D: these constants may vary between individuals and may be altered by pregnancy [25,39,42]; we cannot rule out an effect of the differing affinity constants on the risk for PE. Likewise, vitamin D metabolism may be affected by variation in the allelic forms of VDBP [42,43,44,45,46], but our group sizes were too small for such analyses to be undertaken. We lack the data to evaluate the immunoassay for 1,25(OH)_2_D compared with HPLC. Sunshine exposure affects total 25(OH)D levels, and vitamin D deficiency may have seasonal and geographical variations. In our subset, 51% of the women were from Norway; 29%, from Australia; and 20%, from the USA. The small numbers precluded stratification by season or location, but most participants, regardless of origin, were vitamin D insufficient or deficient. The absence of vitamin D-sufficient women is a limitation.

*Interpretation:* Vitamin D is converted from a pro-hormone, 25(OH)D, to an active hormone by 25(OH)D-1α-hydroxylase, predominantly in the kidney but also in macrophages and, during pregnancy, in the placenta [47]. The higher levels of 1,25(OH)_2_D in diabetic women with subsequent PE could reflect early subclinical renal or placental dysfunction. The kidney is the major site of its formation, and furthermore, VDBP is filtered through the glomerulus and is reabsorbed by the proximal tubules [2,37]. We may hypothesize that early renal dysfunction, prior to PE onset, perturbs the concentrations of 1,25(OH)_2_D and VDBP [48,49].

Women were excluded from our study if they had microalbuminuria or more severe albuminuria at V1. Nevertheless, other evidence from the MAMPED cohort supports the concept that subtle, early renal abnormalities confer PE risk: specifically, increased first trimester urinary neutrophil-gelatinase associated lipocalin (creatinine corrected) (uNGALcc) and elevated estimated glomerular filtration rates (eGFR) [35]. Relating the current data to these prior findings, we observed—specifically in DM+PE+ women at the first trimester—that total, bioavailable, and free 25(OH)D were negatively correlated with eGFR (all *p* < 0.05), while total and free 1,25(OH)_2_D at V2 were positively correlated with uNGALcc (*p* < 0.05). Regarding VDBP, any renal insult during or even before pregnancy could alter glomerular and tubular processing. Overall, these lines of evidence support the notion that subtle subclinical renal dysfunction, preceding microalbuminuria, is associated with PE.

An alternative possibility is that the association between 1,25(OH)_2_D and subsequent PE is “defensive” against stresses initiating disease. For the fetoplacental unit, 1,25(OH)_2_D has protective functions, inhibiting inflammatory cytokines [50] and inducing anti-microbial peptide synthesis [51]. In a rat model (reduced utero-placental perfusion, RUPP), early-gestation treatment with 1,25(OH)_2_D ameliorated PE, apparently by reducing oxidative and ER stresses [6]. Additionally, in a cross-sectional study in non-diabetic humans, plasma 1,25(OH)_2_D was lower in those with than without PE [52], perhaps reflecting the defeat of protective responses.

The associations between vitamin D and PE may differ between “mild, late onset” PE and “early-onset severe” forms of the disease. Bodnar et al. have suggested that the association between vitamin D deficiency and PE is limited to the latter [13]. In the present prospective study, an overwhelming majority (~90%) of PE cases in T1DM women were “mild, late onset”. A prospective study of early-onset, severe disease would need to be much larger, beyond the scope of MAMPED.

Whether any of the associations we have identified reflect a causal relationship and whether vitamin D supplementation might modulate PE risk for women with diabetes are unknown. A recent study showed a marginal reduction in fasting glucose with vitamin D supplementation but was underpowered to address PE [53]. In that study, in contrast to our T1DM patients, the women were largely vitamin D sufficient at study entry. Studies of the effects of vitamin D on human hypertension have yielded generally negative results [54].

*Conclusion:* This is the first longitudinal, observational study to investigate the associations of vitamin D metabolites and VDBP with PE in women with T1DM. In the late second trimester, 1,25(OH)_2_D and the 1,25(OH)_2_D/VDBP ratio were good predictors of PE. Further studies should address the value of these biomarkers, the significance of differential changes in 25(OH)D and 1,25(OH)_2_D during pregnancy, the mechanistic implications, and whether optimizing vitamin D status during pregnancy is effective in reducing the high prevalence of PE in T1DM women.

## Figures and Tables

**Table 1:** Clinical characteristics, at study entry, of women with type 1 diabetes, with and without PE, and normotensive non-diabetic controls.

Clinical Characteristics	DM+PE+ (n = 23)	*p* Value ^a^	DM+PE− (n = 24)	*p* Value ^b^	DM− (n = 19)
Age of woman (years)	28.5 ± 5.6	0.31	29.9 ± 3.8	0.25	31.4 ± 4.5
Ethnicity (%)					
White non-Hispanic	91	0.51	92	0.44	100
Australian	0		4		0
White Australian	9		4		0
BMI (kg/m^2^)	27.9 ± 5.9	**0.028**	24.6 ± 4.1	0.50	23.8 ± 3.8
Smoking (%) ^c^					
No	91	0.69	88	0.55	100
Stopped in pregnancy	5		4		0
Gravida (n)	1.3 ± 0.7	1.00	1.3 ± 0.7	0.19	1.7 ± 1.0
Para (n)	0.2 ± 0.5	0.91	0.2 ± 0.5	0.13	0.5 ± 1.0
Duration of T1DM (years)	16.8 ± 6.8	0.32	14.8 ± 7.0	-	-
HbA1c (%)	7.4 ± 1.2	**0.046**	6.7 ± 1.0	**<0.0001**	5.3 ± 0.3
HbA1c (mmol/mol)	57 ± 14	**0.046**	50 ± 11	**<0.0001**	35 ± 3
Blood pressure (mmHg)					
BP systolic	113.1 ± 12.4	0.26	109.4 ± 9.6	0.23	113.3 ± 8.7
BP diastolic	66.6 ± 9.0	0.27	63.8 ± 8.1	0.24	66.9 ± 7.6
Mean arterial pressure	82.1 ± 9.0	0.21	79.0 ± 7.7	0.14	82.7 ± 6.2
Total daily insulin (IU/d)	62.2 ± 19.7	**0.009**	47.9 ± 14.2	-	-
Total cholesterol (mmol/L)	4.7 ± 0.7	0.53	4.5 ± 0.9	0.18	4.9 ± 0.7
HDL cholesterol (mmol/L)	1.9 ± 0.4	**0.029**	2.2 ± 0.5	0.71	2.1 ± 0.6
LDL cholesterol (mmol/L)	2.4 ± 0.7	0.08	2.0 ± 0.7	0.18	2.3 ± 0.8
Triacylglycerol (mmol/L)	1.0 ± 0.3	0.27	0.8 ± 0.3	0.09	1.1 ± 0.4
Gestational age (weeks)					
Visit 1	12.3 ± 2.1	0.94	12.3 ± 1.7	0.49	12.6 ± 1.7
Visit 2	22.1 ± 1.6	0.18	21.5 ± 1.3	0.95	21.5 ± 1.3
Visit 3	31.7 ± 1.7	0.39	31.3 ± 1.5	0.82	31.2 ± 1.1

Data are presented as means ± SDs. Measurements refer to Visit 1 unless otherwise indicated. Independent sample t tests and *χ^2^* tests were used as appropriate. *p* values <0.05 (statistically significant) are highlighted in bold. ^a^ *p* value, DM+PE− vs. DM+PE+. ^b^ *p* value, DM+PE− vs. DM−. ^c^ *p* values (regarding smoking) refer to comparisons between the sums of percentages in the “no” and “stopped in pregnancy” categories.

**Table 2:** Molar ratios of 1,25(OH)_2_D to 25(OH)D; vitamin D binding protein (VDBP) concentrations; and molar ratios of vitamin D to VDBP.

		DM+PE+					DM+PE−					DM−			
	Visit Number	N	Mean		SD	*p* Value ^a^	N	Mean		SD	*p* Value ^b^	N	Mean		SD
Total 1,25(OH)_2_D/Total 25(OH)D (×10^3^)	1	20	7.2	±	2.6	0.14	23	5.9	±	2.9	0.14	16	4.7	±	1.8
	2	22	6.3	±	2.2	0.99	21	6.3	±	3.0	0.21	18	5.3	±	1.6
	3	19	10.1	±	6.6	**0.021**	23	6.1	±	3.8	0.47	17	5.4	±	2.2
Bioavailable 1,25(OH)_2_D/Bioavailable 25(OH)D (×10^3^)	1	20	11.7	±	4.3	0.15	23	9.7	±	4.9	0.18	15	7.7	±	3.2
	2	21	10.3	±	3.6	0.99	21	10.5	±	4.9	0.21	18	8.9	±	2.7
	3	19	16.9	±	11.2	**0.010**	22	9.6	±	5.5	0.74	17	9.0	±	3.8
Free 1,25(OH)_2_D/Free 25(OH)D (×10^3^)	1	20	127	±	47	0.15	23	105	±	53	0.18	15	84	±	34
	2	21	111	±	39	0.88	21	114	±	53	0.21	18	96	±	29
	3	19	182	±	120	**0.010**	22	103	±	59	0.74	17	97	±	41
VDBP (μmol/L)	1	23	5.3	±	1.1	0.54	23	5.6	±	1.8	0.15	18	6.3	±	1.1
	2	23	6.5	±	0.9	0.95	21	6.5	±	1.2	0.20	18	7.1	±	1.6
	3	23	7.0	±	1.1	**0.032**	24	7.9	±	1.6	0.79	19	8.0	±	1.8
Total 25(OH)D/VDBP (×10^3^)	1	22	8.0	±	4.0	0.42	23	9.0	±	4.5	0.29	18	10.3	±	3.2
	2	22	9.3	±	5.1	0.38	21	8.0	±	4.5	0.15	18	9.9	±	3.7
	3	22	6.9	±	3.3	0.25	24	8.2	±	4.1	0.40	18	9.2	±	3.8
[25(OH)D bound to VDBP *]/VDBP (×10^3^)	1	22	7.2	±	3.5	0.41	23	8.1	±	4.0	0.31	17	9.3	±	2.9
	2	21	8.7	±	4.8	0.36	21	7.4	±	4.2	0.14	18	9.2	±	3.4
	3	22	6.5	±	3.1	0.16	23	7.9	±	3.7	0.52	18	8.7	±	3.5
Total 1,25(OH)_2_D/VDBP (×10^5^)	1	21	4.6	±	0.9	0.33	23	4.3	±	1.0	0.66	16	4.5	±	1.1
	2	23	5.2	±	1.4	**0.005**	21	4.1	±	1.0	**0.025**	18	4.9	±	1.2
	3	20	5.4	±	1.9	**0.005**	23	4.0	±	1.0	0.20	18	4.5	±	1.0
[1,25(OH)_2_D bound to VDBP *]/VDBP (×10^5^)	1	21	3.9	±	0.8	0.33	23	3.7	±	0.9	0.63	15	3.8	±	0.9
	2	22	4.5	±	1.3	**0.007**	21	3.6	±	0.8	**0.018**	18	4.3	±	1.0
	3	20	4.8	±	1.7	**0.009**	22	3.7	±	1.0	0.25	18	4.0	±	0.9

Data are presented as means ± SDs. Independent t tests were used as appropriate. *p* values < 0.05 are highlighted in bold (statistically significant). ^a^ DM+PE− vs. DM+PE+. ^b^ DM+PE− vs. DM−. * Amount bound = total minus bioavailable.

## Data Availability

The data that support the findings of this study are available from the corresponding author on reasonable request.

## Additional Information

A preprint of this manuscript can be found at “Clare Kelly, Carol Wagner, Judy Shary, et al. Vitamin D metabolites and binding protein predict preeclampsia in women with Type 1 diabetes: a cohort study. *Authorea.* May 21, 2020. DOI: 10.22541/au.159007753.32992138”.

## Abbreviations

BMI	Body Mass Index;
DM+PE+	Women with type 1 diabetes who subsequently developed PE;
DM+PE−	Women with type 1 diabetes who did not develop PE;
DM−	Non-diabetic, normotensive women;
eGFR	Estimated glomerular filtration rate;
HbA1c	Glycated hemoglobin;
MAMPED	Markers And Mechanisms for PreEclampsia in Type 1 Diabetes;
MAP	Mean Arterial Pressure;
PE	Preeclampsia;
T1DM	Type 1 diabetes mellitus;
uNGALcc	Urinary neutrophil-gelatinase associated lipocalin (creatinine corrected);
VDBP	Vitamin D Binding Protein;
1,25(OH)_2_D	1,25-dihydroxyvitamin D;
25(OH)D	25-hydroxyvitamin D.
